# Evolving Epidemiology, Improving Diagnostic Tests and Their Importance for the Correct Diagnosis of Histoplasmosis

**DOI:** 10.3390/jof11030196

**Published:** 2025-03-04

**Authors:** Thomas E. Schmidt, Tarsila Vieceli, Lisandra Serra Damasceno, Sarah Kimuda, Alessandro C. Pasqualotto, Nathan C. Bahr

**Affiliations:** 1 Division of Infectious Diseases and International Medicine, Department of Medicine, University of Minnesota, Minneapolis, MN 55455, USA; schm1610@umn.edu; 2 Department of Clinical Medicine, Federal University of Health Sciences of Porto Alegre (UFCSPA), Porto Alegre 90050-170, Brazil; tarsilavieceli@gmail.com (T.V.); pasqualotto@santacasa.org.br (A.C.P.); 3 Department of Community Health, Federal University of Ceara (UFC), Fortaleza 60430-160, Brazil; lisandra.damasceno@ufc.br; 4 Infectious Diseases Institute, Makerere University, Kampala P.O. Box 7072, Uganda; skimuda@idi.co.ug; 5 Infectious Diseases and Internal Medicine Services, Santa Casa de Misericórdia de Porto Alegre, Porto Alegre 90020-090, Brazil

**Keywords:** histoplasmosis, *Histoplasma capsulatum*, diagnostic tests, disseminated histoplasmosis, endemic mycoses, HIV/AIDS

## Abstract

Histoplasmosis has traditionally been described as having discrete geographic areas of endemicity. Over the last few decades, it has become more and more clear that these areas are not accurate depictions of where histoplasmosis can occur. Our understanding of where histoplasmosis occurs has improved in recent years due to improving access to diagnostic testing and increased reporting as well as larger at-risk populations (HIV and non-HIV immune suppression) resulting in more cases. Although areas of relatively higher risk and case numbers certainly still exist, histoplasmosis has been observed in much of the world at this point. Our knowledge of the geographic distribution of histoplasmosis, though improving, remains incomplete. While diagnostic testing has traditionally been confined to visualization and/or culture in much of the world, antigen testing has emerged as an excellent tool. Unfortunately access to antigen testing has been inadequate for much of the world, but this has started to change in recent years and will likely change more dramatically in the near future, assuming ongoing positive developments in the area of lateral flow tests for antigen testing. In this review, we describe the current understanding of the geographic distribution of histoplasmosis, the current landscape of diagnostic testing, and the evolution of both areas with an eye towards the future.

## 1. Introduction

Histoplasmosis is a serious fungal disease that is caused by the fungi *Histoplasma capsulatum* var. *capsulatum* and less commonly by *Histoplasma capsulatum* var. *duboisii. H. capsulatum* is a dimorphic fungus that grows as a mold within the environment and a yeast in humans. The fungi are found in the environment throughout the world and were traditionally thought of as being endemic in Africa, North America, and South America. Recently, cases are increasingly being observed in Asia, Australia, and Europe [1,2,3,4,5,6]. *Histoplasma* species mainly infect humans through inhalation of conidia (fungal spores) from the environment. Of note, additional species of *Histoplasma* have been reported, although their clinical significance in humans is not yet known [7,8]. 

People with immunosuppression have a significant burden of disease compared to immunocompetent individuals. Severe and disseminated infection primarily occurs in people living with HIV/AIDS (PLWHA), solid organ transplant recipients, or those on immunosuppressive medications such as corticosteroids or tumor necrosis factor alpha (TNF-α) inhibitors [2,3,4,5].

Our understanding of the global prevalence of disease is limited by our ability to diagnose the disease. Recently, new data have shed light on where infection due to *H. capsulatum* is occurring outside of areas traditionally considered to be endemic. Clinical suspicion for histoplasmosis is necessary for clinicians to test for the fungi. Symptoms of disease are non-specific and include cough, fatigue, chest pain, and body aches, which can appear 3–17 days after inhalation of spores [9].

Disseminated histoplasmosis (DH) is uniformly fatal when untreated [10]; it is imperative to have diagnostic testing that is rapid, sensitive, specific, and accessible. Our current diagnostic testing strategies for histoplasmosis include direct visualization, histopathology and fungal cultures from sites of concern (lungs, blood, skin, etc.), antigen testing, antibody testing, 1,3 B-D-Glucan, and polymerase chain reaction (PCR) testing. Each of these diagnostics have varying degrees of sensitivity and specificity when used for patients with and without immunosuppression and depending on the syndrome (e.g., disseminated versus limited). Histopathology and direct visualization require highly trained individuals but are perhaps the most widely accessible testing strategies. Fungal culture’s diagnostic accuracy varies with disease burden (as do all tests that detect microbes or their components) and take weeks to return results. Although antigen testing is quicker than culture, it is also dependent on the burden of disease and access is limited in many parts of the world. Antibody testing is reliant upon a functioning immune system and antibodies may not be produced for weeks, even in immunocompetent individuals, limiting their utility in some scenarios. 1,3 B-D-Glucan is a product of many fungi’s cell walls and so is non-specific but may be helpful in some scenarios as a screening test. Lastly, PCR has increasingly been used with varying results depending on the nucleic acid targets and at this point lacks standardization.

In this review, we will discuss the current understanding of histoplasmosis epidemiology, the diagnostics test strategies for histoplasmosis, the effect of immune suppression on both areas, and the future of diagnostic testing and geographic expansion of histoplasmosis. 

## 2. Epidemiology

### 2.1. Overview

Our knowledge of areas thought to be endemic for histoplasmosis has changed rapidly in recent years [11]. Multiple strategies (public health reporting, published data, payor data, skin testing, antibody testing, antigen testing, environmental modeling, etc.) have been used to better understand where histoplasmosis occurs locally and globally. Each of these strategies can be helpful but also has limitations, meaning that unless complete public health data become available globally (with global access to highly sensitive testing and with clinicians having a high clinical suspicion of disease), we will need to consider the data in totality. At present, the data are telling us that histoplasmosis is occurring in most parts of the world but is particularly common in much of North America, South and Central America, as well as large parts of Africa and to a lesser degree (so far) Europe, Asia, Australia, and even Antarctica [12]. While any map is a snapshot in time based on the current data and/or projections, the take home message for clinicians should be that while risk is higher in some locations, any person with a compatible clinical syndrome could have histoplasmosis, regardless of geographic location.

### 2.2. Trends in Latin America

In Latin America, the lack of surveillance of fungal infections in many countries has contributed to lack of the recognition of the true prevalence of this mycosis. Cuba and Nicaragua are the only countries with national surveillance of endemic mycoses. In Venezuela, surveillance is carried out by the National Reference Laboratory, which informs the Ministry of Health [13].

Previous studies using histoplasmin skin testing have identified areas of high prevalence as Guatemala (57.2%), Belize (49.4%), Venezuela (48.2%), and Guyana (47.0%). In Brazil, some areas have a high prevalence of histoplasmosis such as Amapá, Pará, Amazonas, Ceará, Rio Grande do Norte, Paraíba, Pernambuco, Mato Grosso, Rio de Janeiro, São Paulo, and Rio Grande do Sul, where the prevalence ranges between 20 and 50%. In the Caribbean Island and Trinidad and Tobago, the histoplasmin skin test positivity is 42% [2]. Low prevalence was observed in Chile (0.1%), Uruguay (10.2%), and Paraguay (17.5%) [14]. 

In 2017, the total estimated annual incidence of histoplasmosis was ~100,000 cases worldwide [15]. In 2012, the estimated incidence was 1.48 per 100 PLWHA in Latin America, with approximately 22,000–24,000 cases per year of HIV-associated histoplasmosis, similar to the burden of HIV-associated tuberculosis. Guatemala (4.16 per 100 PLWHA), Belize (3.04 per 100 PLWHA), Venezuela (2.90 per 100 PLWHA), and Guyana (2.76 per 100 PLWHA) were the countries with the highest incidence. In addition, high-population countries such as Brazil, Colombia, and Mexico were estimated to have more than 1500 cases per year during the same period. The mortality rate was 40% in PLWHA with LT-CD4+ < 200 cells/μL, resulting in 9600 deaths per year from histoplasmosis, more than from tuberculosis (~6000 deaths) in Latin America [14].

In Colombia, the prevalence of histoplasmosis was 6% in 11,576 samples analyzed in a specialized laboratory [16]. In addition, 349 cases of HIV-associated histoplasmosis were identified in French Guiana between 1981 and 2014 [17]. In Panama, the incidence of HIV-associated histoplasmosis was recently estimated to be 15.1/100,000 PLWHA per year [18]. In Argentina, the annual incidence of DH was estimated to be about 404 cases [19]. 

In Brazil, 3530 cases of histoplasmosis were reported in the literature between 1930 and 2018. About 57% of cases occurred in immunosuppressed individuals, and HIV infection (>97%) was the main underlying condition [20]. Some outbreaks have also been documented in Brazil, mainly associated with cave activity. There are more than 7500 caves in Brazil. Thus, it is important to alert travelers of the histoplasmosis risk during ecotourism activities on these sites [21]. As this condition is not notified to health authorities in Brazil, the true incidence of histoplasmosis in Brazil is unknown. This is a sadly typical situation in many countries in Latin America but also throughout the world.

Many countries have seen an increase in the number of histoplasmosis cases in advanced HIV patients since access to antigen testing through screening of people with CD4+ T-lymphocytes < 200 cells/μL. A recent meta-analysis of antigen detection performance from 18 studies showed an overall sensitivity of 95% and specificity of 97% in PLWHA [22]. 

In Guatemala, the incidence of HIV-associated histoplasmosis increased from 6.5% in 2017 to 8.8% in 2019 due to screening with antigen testing [23]. In other countries such as Argentina, Paraguay, Nicaragua, Honduras, and Panama, the positivity of antigen detection ranged from 9 to 32% in patients with advanced AIDS [24,25,26]. In Brazil, antigen testing increased the diagnosis of histoplasmosis in PLWHA by more than 50% in a large cohort compared with conventional methods [27]. A recent systematic review found a prevalence of *Histoplasma* antigenuria in Latin America ranging from 3.8 to 21.5% [28]. In addition, antigen detection has also demonstrated excellent performance in disseminated histoplasmosis post-transplantation, with a sensitivity of 93% in the cohort of 142 patients [29]. 

Cases have been reported in other countries within the region including a number of cases in Peru and a single case purported to have occurred due to risk in Bolivia, but countrywide or larger regional prevalence data are not available [30,31,32].

Outside the USA, the data on histoplasmosis in non-HIV populations are minimal. In Brazil, there are few cases of post-transplant histoplasmosis, and the incidence rate ranges from 0.2% to 1.1% [33,34]. In Mexico, 50 cases of histoplasmosis in non-HIV patients were identified. The most common underlying diseases identified were rheumatic diseases, chronic kidney disease, and renal transplantation [35]. In French Guiana, 31 non-HIV cases in adults and 3 in children were identified between 2008 and 2022 [36]. From 1939 to 2021, approximately 1461 cases of pediatric histoplasmosis were diagnosed, with 97.1% occurring in non-HIV individuals. Most cases were identified in North and Central America, with 1231 cases. Malignancy, pancytopenia, T-cell deficiency, hyperimmunoglobulinemia, organ transplantation, malnutrition, and autoimmune diseases were the main conditions associated with histoplasmosis. Many cases were diagnosed by serology [37]. In Colombia, 45 pediatric cases were identified. The main risk factors were malnutrition and environmental exposure [38]. This is another area where, with improved access to antigen testing (and increasing numbers of persons with non-HIV immune suppression), numbers are likely to increase in areas with high incidences of histoplasmosis in PLWHA.

### 2.3. North America

The United States is a highly endemic country for histoplasmosis, with very high rates in the Mississippi and Ohio Valley regions but also with cases being reported throughout much of the country [11,39]. Based on state-specific incidence rates, between 2011 and 2014, the incidence rate ranged from 0 to 4.3 cases/100,000 population, and a stable trend was observed, although these data are limited to states that report histoplasmosis [40]. Yet, in recent years our knowledge of the geographic distribution of histoplasmosis has expanded due to climate change, advanced HIV disease, and other immunosuppressive conditions beyond HIV infection [39]. 

Among 13 U.S. states that conducted epidemiological surveillance for histoplasmosis [41], a decrease in AIDS-associated histoplasmosis has been observed in hospital admissions. In 2001, 21.5% of AIDS patients were hospitalized due to histoplasmosis, compared to 17.3% in 2012 [42]. At a tertiary care center in Kentucky, the proportion of HIV-associated histoplasmosis decreased from 67% in 2000–2001 to 18% in 2008–2009. Access to antiretroviral therapy has been associated with a proportional decrease in this mycosis in PLWHA [43]. 

On the other hand, histoplasmosis also occurs in patients with other immunosuppressive conditions such as autoimmune diseases, chronic pulmonary obstructive disease, asthma requiring an inhaler, heart disease, people who use TNF-α inhibitors, and cancer [44,45,46]. Between 2001 and 2006, 15 centers of the Transplant-Associated Infection Surveillance Network registered 52 cases of histoplasmosis, of which 48 occurred in solid organ transplant recipients and 4 in hematopoietic cell recipients [47]. Another cohort of 24 solid organ transplant (SOT) centers observed 152 cases of histoplasmosis between 2003 and 2010 [29]. 

Between 2007 and 2016, a large retrospective cohort study based on medical claims data registries identified 79,749 cases of histoplasmosis in people aged ≥65 years. About 90% of U.S. states reported histoplasmosis cases, and 62% of U.S. counties had more than 5 histoplasmosis cases. Areas traditionally thought not to be endemic for histoplasmosis such as California, Arizona, Montana, Washington, Alaska, and Hawaii recorded cases of this mycosis [11]. 

From 2019 to 2021, during the COVID-19 pandemic, one publication described 3595 cases of histoplasmosis in states where histoplasmosis is reported to the U.S. Centers for Disease Control and Prevention. The incidence ranged from 1.4 to 2.0 per 100,000 persons. Most cases occurred in people aged 40–64 years. The year 2020 was the year with the lowest number of cases, possibly reflecting lower testing given the massive (and understandable) focus on COVID-19 [48].

In Canada, Ontario and Quebec are now known to be endemic areas for histoplasmosis [49]. Histoplasmosis case registries are not mandatory in Canada although epidemiological surveillance is carried out in some provinces. Between 1990 and 2015, 211 cases of histoplasmosis were diagnosed by isolation of *H. capsulatum* in Ontario [50]. However, other regions such as Alberta and Saskatchewan have reported autochthonous cases of histoplasmosis [51]. As noted above, histoplasmosis is commonly seen in Mexico.

### 2.4. Africa

Africa has seen a steady rise in the number of histoplasmosis cases reported following an increase in medical mycology advocacy and clinician sensitization to the presence of a number of fungal infections [52]. In the last six decades, there have been 470 histoplasmosis cases reported in Africa, 38% were among PLWHA [52]. This is almost certainly a vast underestimation due to reporting bias, a lack of diagnostic test availability, and low index of clinical suspicion. Kwizera et al., reviewed the number of histoplasmosis cases reported over 70 years in Uganda and found that out of 64 cases, only eight cases were correctly diagnosed clinically and histopathologically [53]. There are two variants responsible for histoplasmosis infection on the African continent, *Histoplasma capsulatum* var. *duboisii* and *Histoplasma capsulatum* var *capsulatum* [52]. *Histoplasma capsulatum* var. *duboisii* has been more commonly reported and is found mainly in West and Central Africa. The few cases of *Histoplasma capsulatum* var. *duboisii* reported outside of Africa were amongst individuals with past travel history to Africa [54]. *Histoplasma capsulatum* var *capsulatum* has been mostly reported in Southern Africa although with some cases in North and East Africa as well. However, it has been posited that some of the cases reported in Southern Africa might have actually been *Emergomyces africanus* [55]. The cases could also have been imported cases as South Africa has a large number of non-African immigrants. A history of exposure to nitrogen-rich guano soil and bat droppings has been identified as a risk factor for histoplasmosis infection with a number of cases identified in individuals such as cave guides, those that climb bat trees [56], and sawmill workers as seen in Uganda [54].

PLWHA are more likely to have a co-infection with *Histoplasma capsulatum* var *capsulatum* as it is the type that affects immunocompromised patients (versus *Histoplasma capsulatum* var *duboisii*) [52]. Persons with HIV and histoplasmosis co-infection are more likely to have poor outcomes due to delayed or missed diagnosis. In many areas, part of the delay in diagnosis is that there is a similarity in symptoms with pulmonary tuberculosis (TB) and as a result, many are conferred a diagnosis of presumptive pulmonary TB but actually have histoplasmosis infection [54]. Unfortunately, due to the gaps in diagnosis, some patients with HIV are only diagnosed with histoplasmosis at postmortem [57]. Cutaneous lesions in patients with HIV that are pathognomonic for histoplasmosis may be misdiagnosed as cryptococcosis or molluscum contagiosum [54]. These issues combined mean that it is quite likely that histoplasmosis is underestimated currently in much of Africa. There has not been a significant drop in the number of histoplasmosis cases with the rollout of antiretroviral therapy [52].

The reported prevalence of histoplasmosis across the African continent varies according to region. In Uganda, among 157 samples from persons with advanced HIV presenting with cryptococcal meningitis, blood *Histoplasma* antigen was not detected in any cases [58]. Yet, a subsequent study did find positive urine antigen tests in 1.2% (4/388) Ugandan outpatients with advanced HIV [59]. On the other hand, in West and South Africa, prevalence rates were higher, notably with varying proportions of people with HIV among the study populations. *Histoplasma* antigenuria incidence was 26% in Cameroon [54]. In one study in Nigeria where 19.2% of the patients had advanced HIV disease, the incidence of Histoplasma antigenuria was found to be between 7.7 and 12.7 [54,60]. In Ghana, the incidence was 4.7% amongst patients with HIV disease and this was irrespective of HIV disease stage [61]. Amongst patients with advanced HIV disease in South Africa, the incidence was found to be 14% [62]. The diagnosis of histoplasmosis has evolved over the years from the use of culture, histopathology, serology, cytology, to antigen testing currently (although the latter tests still remain useful in some situations, including if antigen testing is not available). *Histoplasma* antigen testing will improve diagnosis of disseminated histoplasmosis in the acute setting and reduce time to diagnosis and treatment [63]. However, tests available in this setting, such as the IMMY EIA urine test, are limited by the fact that they are designed to only detect the galactomannan antigen for the variant *Histoplasma capsulatum* var *capsulatum* [64]. As a result, cases of *Histoplasma capsulatum* var *dubuosii* could be missed [28]. Overall, there is still a need to increase awareness amongst health personnel of histoplasmosis, and the need to consider the diagnosis.

### 2.5. Areas Traditionally Thought Not to Be Endemic: Europe, Asia, Oceania, and Antarctica

Although histoplasmosis is understood to occur in the Americas and Africa, non-endemic areas, including Europe, Asia, and Oceania, have reported an increasing number of cases due to global travel, migration, immunocompromised populations, and higher suspicion of the disease. The under-recognition of histoplasmosis in these regions, coupled with limited diagnostic capacity, poses challenges for timely identification and treatment.

### 2.6. Europe

Histoplasmosis is rare in Europe, with most cases linked to travel to endemic regions, primarily in Latin America and Africa. Cases have been documented in countries such as Italy, Spain, and Switzerland, with immunocompromised patients being particularly susceptible. Reactivation of the disease up to 50 years after the initial infection has been documented, as has transmission of infection by transplant donors to recipients [65]. In Italy, histoplasmosis has been recognized in travelers returning from endemic regions, where exposure to bat and bird droppings in caves or construction sites is common [66]. Yet autochthonous cases have been noted as well, such as a severe case of pulmonary histoplasmosis in Switzerland, suggesting potential environmental reservoirs [1]. Similar cases have been reported in Italy.

### 2.7. Asia

Asia has emerged as a region with a growing number of histoplasmosis cases, particularly in India and China. In India, histoplasmosis is increasingly recognized in patients with adrenal involvement or disseminated disease and is often misdiagnosed as tuberculosis due to overlapping clinical features. A study from Himachal Pradesh noted several cases of DH in HIV-negative adults, highlighting the importance of including this fungal infection in the differential diagnosis of pyrexia of unknown origin [67].

In China, while histoplasmosis remains relatively rare, increased reporting has led to an expanding geographical distribution. A retrospective analysis of 101 cases in mainland China demonstrated that most cases were concentrated in southern China, although northern regions also have reported cases [6,68,69]. In immunocompromised patients, such as those undergoing organ transplantation, pulmonary histoplasmosis is frequently misdiagnosed as cancer or tuberculosis. Japan has also reported an increase in the number of histoplasmosis cases, with most patients having reported time spent in South American countries [70]. A systematic review of reported cases of histoplasmosis in Asia found a number of cases in Thailand, Malaysia, Indonesia, and Singapore; almost half occurred in PLWHA. Additionally, the authors found that high histoplasmin skin test prevalence was found in Myanmar, the Philippines, Indonesia, Thailand, and Vietnam [71].

### 2.8. Oceania

In Oceania, Australia is the primary country reporting histoplasmosis cases, with most infections linked to travel or specific local environments, such as caves inhabited by bats [72]. Histoplasmosis in Australia is predominantly found in Queensland and New South Wales, with both acute pulmonary and disseminated forms reported [73]. Several outbreaks have been linked to cave exploration, specifically at the Church Cave system, where bat guano serves as a reservoir for *H. capsulatum* [73]. In one notable case series, 16 cases of histoplasmosis were documented, many of which were associated with immunosuppressed patients, particularly those living with HIV [73]. Autochthonous cases in Queensland further underscore the need for increased awareness of histoplasmosis in regions previously not considered endemic.

While environmental sources of *H. capsulatum* in Australia remain obscure, soil samples contaminated with bat and bird droppings in specific regions have been identified as potential reservoirs [74]. Given the increasing number of cases, there have been calls to introduce antigen detection assays to improve diagnostic accuracy.

In conclusion, histoplasmosis is increasingly recognized in non-endemic regions, including Europe, Asia, and Oceania, driven by globalization and the rise of immunocompromised populations. We now know that *Histoplasma* can also be found even in Antarctica [12]. Despite its growing incidence, the disease remains underdiagnosed due to a lack of clinical awareness and easily accessible diagnostic tools. There is an ongoing need for improved epidemiologic understanding of disease distribution which would be aided significantly by increased public health reporting requirements. To improve patient outcomes, healthcare providers, regardless of whether they live in a region of known endemicity (noting risk is not uniform and some areas are clearly higher risk), must become more vigilant, particularly with immunocompromised individuals. Expanding access to rapid diagnostic tools, such as antigen detection will be essential to address the rising burden of histoplasmosis in traditionally ‘non-endemic’ areas.

### 2.9. The Impact of HIV on Histoplasmosis Epidemiology

Persons with HIV, especially those with CD4 counts below 150 cells/µL, are at heightened risk for severe forms of histoplasmosis, often leading to disseminated disease. HIV patients in Latin America have a greater risk of histoplasmosis than TB when CD4 counts are extremely low. The compromised immune system in these patients allows *H. capsulatum* to disseminate beyond the lungs, resulting in multi-organ involvement, severe morbidity, and high mortality if left untreated. Common findings include fever, weight loss, hepatosplenomegaly, pancytopenia, and respiratory distress, mimicking other infections like TB, which often leads to misdiagnosis [27].

In Latin America, where HIV and *Histoplasma* overlap geographically, histoplasmosis has emerged as the leading cause of death in AIDS patients. In contrast to TB, which is widely recognized and tested for, histoplasmosis remains underdiagnosed due to limited diagnostic resources and clinical awareness [22]. In regions like these, histoplasmosis antigen detection in urine and serum has emerged as a critical diagnostic tool to identify the disease early, especially in severely immunosuppressed patients [75]. The antigen detection method is highly sensitive for disseminated histoplasmosis, particularly in HIV-infected individuals, allowing for earlier diagnosis and treatment compared to traditional culture-based methods. 

It is important to note that high frequencies of co-infection with histoplasmosis and tuberculosis in PLWHA are observed in Panama (38%), Colombia (35%), Guatemala (26%), and Brazil (26%). Importantly, as the CD4 count decreases, the likelihood of histoplasmosis rather than TB being the cause of illness rises in areas with high rates of histoplasmosis infections [72]. In this context, the antigen test can also help to diagnose histoplasmosis in persons with or without tuberculosis [22]. This diagnostic challenge underscores the importance of histoplasmosis antigen detection, particularly in resource-limited settings where other diagnostic modalities may not be readily available. Improving access to these diagnostic tests in endemic regions is essential to reduce mortality from histoplasmosis in HIV-infected populations and will also improve our understanding of where histoplasmosis occurs [76].

### 2.10. Non-HIV Immunosuppression and Histoplasmosis Epidemiology

Non-HIV-related immunosuppression also plays a significant role in the epidemiology of histoplasmosis. Patients undergoing organ transplantation, cancer therapy, or long-term corticosteroid use are at increased risk for histoplasmosis, including disseminated disease.

In cancer patients, especially those undergoing chemotherapy for hematologic malignancies, histoplasmosis can be severe and life-threatening. The disease often presents atypically in these patients, mimicking lung cancer or other malignancies, further complicating the diagnosis [77]. The use of histoplasmosis antigen detection in these populations can facilitate early diagnosis, as it provides rapid, non-invasive testing that is particularly effective for disseminated disease.

Corticosteroids that are commonly used to treat persons with autoimmune diseases, those requiring organ transplantation, among others, can also predispose individuals to histoplasmosis. Patients receiving long-term corticosteroid therapy are at increased risk for severe DH, especially in hyperendemic regions [49].

The increasing use of biologic agents, such as TNF-α inhibitors, has also contributed to the rising incidence of histoplasmosis in immunosuppressed patients [45,78]. TNF-α is crucial for the immune response to *H. capsulatum*, and inhibition of TNF-α can impair the body’s ability to control fungal infections, leading to increased susceptibility to DH. Patients on TNF-α inhibitors for conditions such as rheumatoid arthritis and inflammatory bowel disease are at particular risk in endemic areas.

The increased use of immunosuppressant agents in these patient groups and others had led to an increased proportion of the population at risk for histoplasmosis and ultimately, increased infections. In some cases, this scenario has also led to awareness of the potential for histoplasmosis in areas where histoplasmosis was not previously thought to occur.

## 3. Diagnostic Testing

### 3.1. The Current State of Diagnostic Testing

The EORTC (European Organization for the Research and Treatment of Cancer)/MSGERC (Mycoses Study Group Education and Research Consortium) have defined criteria for the diagnosis of histoplasmosis: a proven diagnosis comprises confirmation by either histopathology or culture and a probable diagnosis is based on the presence of clinical presentation, predisposing condition, and mycological evidence, such as antigenuria [79]. 

The isolation of *H. capsulatum* for clinical specimens remains the gold standard for histoplasmosis diagnosis; it was used to first describe the fungus in 1906 by Darling [80]. Serologic tests, such as complement-fixation, have been employed since the 1950s, and the implications of a positive result on defining past or current infection by *H. capsulatum* has been widely discussed in the past century [81,82,83]. Histopathology has been used for over 70 years for diagnosis of histoplasmosis in both humans and animals [84,85] and specific staining, such as Methenamine Silver and Periodic Acid-Schiff (PAS), as well as the intracellular behavior of the fungus, have been described since the 1960s [86]. 

More recently, *H. capsulatum* antigen detection assays were developed [87], revolutionizing the diagnosis of the disease, as this method does not rely on samples obtained through invasive tests or require weeks before results as fungal cultures do. MiraVista (MVista, Indianapolis, IN, USA) first developed an *H. capsulatum* quantitative enzyme immunoassay (EIA) followed by subsequent generations of the test. Unfortunately, these tests have largely been unavailable commercially outside of the United States. Within the United States, these tests are sent to a single laboratory, adding to turnaround time. IMMY (Norman, OK, USA) subsequently created the first *Histoplasma* antigen test able to be used beyond the United States, the IMMY ALPHA ELISA kit, a polyclonal antibody immunoassay, validated in 2007 [88]. The Centers for Disease Control (CDC) later developed an in-house enzyme-linked immunosorbent assay (ELISA) for *H. capsulatum* detection, which is no longer produced. Years later, IMMY introduced a *Histoplasma* antigen detection test utilizing monoclonal antibodies (Clarus assay), which demonstrated enhanced sensitivity in comparison with their polyclonal assay which is no longer in use [89,90]. PCR tests were later developed but are not commercially available at this time [91] and the use of (1→3)-β-D-Glucan in histoplasmosis diagnosis has been described over the past two decades [92,93].

Accuracy of different diagnostic methods for histoplasmosis depends on the burden of disease, host immunity, and clinical syndrome. Urinary antigen detection is more useful in patients with DH; serology sensitivity can be very low in immunosuppressed patients that may not mount antibody response [94], but it can be useful in patients with subacute or chronic histoplasmosis, where antigen performance is poor [95]. Serum (1→3)-β-D-Glucan is a useful diagnostic tool particularly in patients with DH, but specificity is low. Table 1 describes diagnostic accuracy measures of the various testing modalities as well as their strengths and weaknesses.

Although there are differences in performance that would justify the selection of a particular test for diagnosis, in many regions of the globe, access to diagnostic tools serves as the primary determinant in choosing which test to utilize. Advances in non-culture-based diagnostics have not reached most low and middle income countries; in a recent survey performed in 21 African countries, only 35% of medical centers had access to antigen testing, most of them through a reference laboratory [107]. Histological examination remains the most used technique for histoplasmosis diagnosis in the African continent [108], and only 22% of medical centers in Latin America and the Caribbean report having access to *Histoplasma* urinary antigen [109].

### 3.2. Culture and Pathologic Methods

Culture on Sabouraud’s dextrose agar is incubated at 25 °C to promote the growth of the mycelial phase of *H. capsulatum*. After up to 6 weeks, a white to light tan mold develops and a lactophenol cotton blue test can be performed to determine morphology. The hyphae produce two types of conidia: macroconidia, also known as tuberculate conidia, measuring 7–15 μm in diameter and featuring distinctive surface projections; and microconidia, which are smaller (2–5 μm) and have smooth walls. The presence of tuberculate macroconidia can lead to a presumptive diagnosis of histoplasmosis, but it is important to note that fungi from the genus *Sepedonium* can also produce similar tuberculate macroconidia [94,110]. 

Accuracy of fungal culture does not seem to vary according to immune status (overall, with some variability between studies), but it does vary widely among clinical forms of the disease [96]. Sensitivity is very low (around 40%) for acute pulmonary histoplasmosis (APH); it is higher for patients with chronic pulmonary (67%) and disseminated (74%) forms of disease. In these patients, the highest yield for culture is obtained using bronchoalveolar lavage (BAL) and bone marrow samples, respectively [96]. Sensitivity is very low for cerebrospinal fluid (CSF) samples [111], imposing a challenge for diagnosis of cerebral histoplasmosis in settings where non-culture-based methods are not available. Manipulation of *H. capsulatum* in fungal cultures requires a laboratory with biosafety level 3 or above for the filamentous form of the fungus; nevertheless, in lower resource settings, fungal culture is sometimes managed in biosafety level 2 laboratories, but this is not recommended and does put laboratory personnel at risk.

Histological examination does not depend on waiting for fungal growth; however, it usually requires invasive tissue samples. The presence of caseating or non-caseating granulomas is typical of histoplasmosis, although this feature is shared by a number of other clinical conditions, such as tuberculosis and sarcoidosis. Hematoxylin-eosin (HE) is insufficient and specific stains, such as PAS and Gromori–Groccott, are usually required for diagnosis [97,112].

The demonstration of yeast cells in tissue supports histoplasmosis diagnosis. Yeasts are ovoid, measure 2–5 μm, and have narrow base budding; they are usually found within macrophages and histiocytes. The differential diagnosis is wide and includes *Cryptococcus, Blastomyces, Candida, Pneumocystis, Coccidioides, Talaromyces, Leishmania, Toxoplasma,* and *Trypanosoma*. *H. capsulatum* var *duboisii* has larger yeasts (8–15 μm) and can demonstrate larger base budding, possibly leading to misdiagnosis as *Blastomyces dermatitidis* [101].

The sensitivity of this method is very low for pulmonary forms of histoplasmosis and is best in patients with high fungal burden, such as those with AIDS or other forms of severe immunosuppression and DH [89]. Detecting *H. capsulatum* yeasts in the presence of a compatible clinical syndrome indicates active infection. Nevertheless, it is important to note that nonviable organisms might be detected for years after infection [95], especially in lymph nodes or lung tissue where calcification can indicate prior infection [112].

Matrix-assisted laser desportion/ionization time of flight mass spectrometry (MALTI-ToF MS) can be used to correctly identify Histoplasma capsulatum yeast forms and early mycelial cultures, where available [113].

### 3.3. Antigen Testing

In countries where *Histoplasma* antigen detection is widely available, it has become a leading modality in histoplasmosis diagnosis. Sensitivity and specificity for antigen detection in urine is approximately 95% and 99% in DH in PLWHA; sensitivity is slightly less for blood antigen detection [98,114]. Antigen tests are less sensitive in chronic pulmonary (87%) and acute pulmonary histoplasmosis (69–83%) [99]. Sensitivity is lowest for patients with subacute histoplasmosis (around 30%) [97].

Antigen accuracy is generally higher in patients with some form of immunosuppression—this is due to higher fungal burden in these patients. In a cohort of patients with DH, antigen sensitivity was 94.6% in patients with AIDS, 93.1% in patients with other immunosuppression, and 73.3% in patients without immunosuppression [96]. For patients with pulmonary forms, the highest yield for antigen detection is achieved by testing both urine and serum. In a study with patients with APH, antigen was detected in 83% of patients by testing both urine and serum, of which 45.8% had only antigenemia [99]. The added yield from urine alone to urine and serum is more incremental.

Antigen has been tested in other body fluids, such as BAL and CSF. In patients with HIV/AIDS and pulmonary histoplasmosis, BAL sensitivity is 70 to 93% compared to 79 to 93% in urine and 65 to 88.5% in serum [115]; BAL fluid may detect cases missed by serum/urine antigen. In patients with *Histoplasma* meningitis, CSF antigen sensitivity ranges from 40 to 78% [97,116], providing an important diagnostic advancement considering the low yield of fungal cultures in CSF and the impossibility of performing histopathology in patients with this condition without requiring invasive tissue biopsy.

Currently, two enzyme immunoassays (EIAs) are widely used for *Histoplasma* antigen detection: MiraVista (MVista) *H. capsulatum* quantitative EIA and IMMY EIA, with high agreement among these tests, (97.6%) [100]; although there is a trend towards lower sensitivity and specificity with IMMY EIA [117]. Both tests have been utilized in patients with HIV/AIDS and DH, with excellent accuracy; a double-blind study evaluating both antigen tests in the diagnosis of DH in PLWHA found a 91.3% sensitivity and 90.9% specificity for IMMY versus 90.4% sensitivity and 92.3% specificity for MVista EIA [118]. More recently, Optimum Imaging Diagnostics (OIDx) has developed a lateral flow assay and Gotham Biotech has commercialized a *Blastomyces* EIA for the detection of *Blastomyces* and *Histoplasma* antigen; data are more limited for these two assays [61,119,120]. In the case of the OIDx assay, more data have begun to emerge. While specificity appears to be less than other antigen assays, more data are needed.

A notable constraint of antigen detection is cross-reactivity with antigens from other fungi, such as *Blastomyces dermatitidis, Paracoccidioides brasiliensis, Talaromyces marneffei*, and, to a lesser extent, *Coccidioides spp* and *Sporothrix schenkii* [97]. Furthermore, false-positive results have been observed in approximately 15% of transplant recipients undergoing anti-rejection therapy with anti-thymocyte globulin [95].

Overall, antigen testing provides fast and reliable results for histoplasmosis diagnosis. Additionally, it has been shown to be useful in monitoring response to therapy, even though antigen titers decrease slowly after antifungal therapy. Antigen levels, particularly in serum, have been demonstrated to decrease in response to effective treatment and to increase in cases of treatment failure, thus serving as a valuable indicator of therapeutic response. The monitoring of antigenemia and antigenuria has been more widely studied in patients with HIV/AIDS; in this population, urine and serum antigen levels of less than 2 ng/mL have been proposed as criteria for establishing a cure and for the discontinuation of antifungal therapy [43]. Importantly, antigen kinetics have been less well described with newer generation assays and so whether any particular cut-off or approach to antigen monitoring is used at this time is more related to convention than current evidence. 

### 3.4. Antibody Testing

Antibodies against *H. capsulatum* are produced 4–8 weeks after infection and may persist for years. Therefore, serology is not useful in the diagnosis of acute forms of histoplasmosis; nevertheless, it provides reasonable accuracy in patients where antigen performance is suboptimal: sensitivity is around 95%, 83%, and 54% in patients with subacute pulmonary, chronic pulmonary, and mediastinal histoplasmosis, respectively [97]. 

Currently, three methodologies are commonly used for histoplasmosis diagnosis: immunodiffusion (ID), complement fixation (CF), and enzyme immunoassay (EIA); the former two are more commonly used. As it occurs with other serologic tests, a positive antibody test for *H. capsulatum* indicates that the patient has had previous contact with the fungus. However, in some scenarios, serologic testing may provide evidence of acute infection. 

The CF method detects the extent of complement fixation to complexes of antibodies with yeast-phase and mycelium-phase antigens. A fourfold rise in CF antibody titers taken at least 2 weeks apart or the detection of *H. capsulatum* antibodies by single CF titer of ≥1:32 are suggestive of recent infection. Low positive results (≥1:8) indicate previous contact with the fungus and may occur in up to a third of patients with active histoplasmosis. Titers usually decrease, although not always completely, with resolution of disease and generally take years to do so [95,97]. Cross-reactivity is observed with other clinical conditions (such as tuberculosis and sarcoidosis) and fungal infections, such as blastomycosis, candidosis, and paracoccidioidomycosis. 

The ID method is based on the precipitation of anti-M and anti-H antibodies. H precipitins are present in less than a quarter of patients and usually clear within 6 months of infection. M precipitins, present in 75–80% of individuals who had been infected, remain positive for many years and, therefore, cannot differentiate acute from prior infection. ID confirms acute histoplasmosis when the H band is present or when there is seroconversion of the M band. It is more specific, albeit less sensitive, than CF [121]: although specificity of ID methods is around 83–100% [96,102], and sensitivity is very low, particularly in immunosuppressed patients. A meta-analysis including only patients with HIV found a sensitivity of 58% for various types of antibody detection [114].

Antibody detection by EIA is based on the detection of anti-*Histoplasma* IgM and IgM antibodies by EIA. It is more sensitive than both ID and CF. For patients with APH, sensitivity is around 67% for IgM and 87.5% for IgG antibodies [103]. However, it is not as available as the other methodologies.

Serology testing can be particularly useful in patients with *Histoplasma* meningitis. The presence of antibodies in CSF by CF, ID, or EIA is sufficient for diagnosis and has higher sensitivity in comparison with fungal cultures: sensitivity of CF or ID detection in CSF is around 51% with 96% specificity. When using EIA, up to 82% of patients with *Histoplasma* meningitis may have positive IgM or IgG antibodies in CSF [116].

Combining serology with antigen testing has been shown to improve the diagnostic yield for APH and *Histoplasma* meningitis [94,116].

### 3.5. PCR

Histoplasmosis PCR tests have been developed with the goals of decreasing time to diagnosis, increasing sensitivity, and increasing specificity. For these platforms to be successful, they need to utilize molecular targets that are specific to *Histoplasma* and be conserved within the fungus. Multiple groups have studied various molecular targets within the universal fungal region, ribosomal RNA, H antigen, M antigen, and more. 

In one Brazilian study conducted assessing 14 patients coinfected with HIV and histoplasmosis, PCR testing on blood specimens was 85.7% sensitive [104]. Additionally, in a French study of 44 patients diagnosed with histoplasmosis, 97.7% were positive by PCR testing on either BAL or blood [105]. Immunosuppression has not been shown to affect PCR testing at this time. A group in Germany showed a clinical sensitivity of 94% with a specific Histoplasma qPCR and 48.5% with a 28s PCR approach on formalin-fixed paraffin-embedded tissues samples from proven histoplasmosis diagnosis. Specificity was 100% on the Histoplasma qPCR platform. A study by Sturny-Leclère et al. on 106 PLWHA demonstrated the potential for PCR testing of blood to determine *Histoplasma* fungal burden and early response to treatment. In patients with DH, there was an association between progression or stabilization of *Histoplasma* burden during treatment and early death at Day 7 and Day 14 [122].

Despite these encouraging studies, challenges remain as there is no standardization in protocols, the molecular targets vary, and the tissue/fluid tested varies. At this time, there are no FDA-approved commercially available PCR diagnostic tests available. Notably, PCR can also be used to rapidly identify culture isolates of *Histoplasma* spp. This has particular value in areas where there is less experience morphologically identifying *Histoplasma*.

### 3.6. Beta D Glucan

1,3 beta-*D*-glucan is a component of the cell wall of most clinically important fungi and can be measured in serum or CSF [123]. In 2008, Egan and colleagues evaluated serum 1,3 beta-*D*-glucan on serum from 23 patients with presumed histoplasmosis and found 87% sensitivity but only 68% specificity among 22 controls [92]. A 2021 study found a sensitivity of 92% (33/36) among people with HIV and DH [106]. The study included only people with *Pneumocystis jirovecii* infection or histoplasmosis and so could not inform specificity more generally. A 2022 study had similar sensitivity in proven (e.g., positive by culture or visualization) DH (88%, 21/24) but was positive in no cases of pulmonary histoplasmosis [93]. Lastly, a study of 1,3 beta-*D*-glucan in CSF for *Histoplasma* meningitis found sensitivity of 53% (25/47) and specificity of 87% (133/153) [63]. Overall, 1,3 beta-*D*-glucan has reasonable sensitivity for DH but should not be used in place of more sensitive antigen testing or culture. Further, a positive 1,3 beta-*D*-glucan cannot definitively diagnose histoplasmosis and more specific testing is needed. These concepts are particularly true when testing CSF where 1,3 beta-*D*-glucan performance is poorer for histoplasmosis. 

## 4. The Future of Diagnostic Testing for Histoplasmosis

As we look at an increasingly known global burden of histoplasmosis due to greater testing, increased at-risk populations for disseminated disease, and increased awareness, it is imperative that we improve our approach to diagnosis. While our gold standard for histoplasmosis testing has been fungal culture and histopathology, these are both time intensive as cultures can take weeks before becoming positive. Histopathology requires highly trained individuals to make the diagnosis and review the specimens. Obtaining these samples can be invasive and burdensome.

Increased focus has been on non-invasive testing such as antibody, antigen, PCR, and 1,3 B-D-Glucan. These tests, however, vary in their sensitivity and specificity depending on the site of infection and the fungal burden. Advancements in antigen testing are in the pipeline now as there is movement to develop lateral flow assays that can give rapid results and require minimal laboratory infrastructure and could drastically decrease the turnaround time from obtaining a sample to diagnosis. OIDx and MiraVista have developed LFAs, both have European Union Conformite Europeenne (CE) marks but no U.S. FDA approval. The OIDx LFA showed 98.4% concordance with the IMMY EIA among 150 urine samples from PLWHA (a mix of symptomatic and asymptomatic patients) and while the OIDx test did have positive results in all five tests positive by EIA, there was one additional false positive test [61]. Another study in Trinidad analyzed 280 samples from PLWHA and a CD4 < 350 cells/mcL, 6.4% (18/280) persons had Histoplasma antigen detected, the OIDx assay sensitivity and specificity were 88.9% (95% CI, 65.3–98.6%) and 93.9% (95% CI, 90.3–96.5%), respectively [124]. Lastly, among 204 persons with suspect histoplasmosis (low HIV prevalence and a mixture of pulmonary and disseminated disease), sensitivity was 33.3% (3/9, 95% CI 7.5–70.1%) and specificity 80.5% (157/195, 95% CI 74.3–85.8%) in proven histoplasmosis, the MVista EIA had equal sensitivity but better specificity (96.9%) [120].

MiraVista has also developed an LFA where sensitivity in serum from 75 PLWHA (21 with culture-proven DH and 51 with other fungal or bacterial infections) showed a sensitivity of 96% and specificity of 90% [125]. In urine, among 352 participants including 44 proven and 22 probable cases of histoplasmosis (with 286 controls) and of which 71% had immune compromise and cases were roughly ⅓ pulmonary and ⅔ DH, sensitivity and specificity of the LFA were 78.8% and 99.3%, respectively [126]. As expected, sensitivity was higher in disseminated disease than pulmonary disease. Additional studies exist [118,127,128]. Among published studies, generally, the performance is, as expected, best in those with DH and advanced HIV and less among people with pulmonary disease. 

Both LFAs (MiraVista and OIDx) with published data [118] do have cross-reactions that occur with other fungi as did prior antigen detection assays. IMMY is also developing an LFA, but this assay has not yet been clinically tested. Further study is needed to precisely determine the diagnostic accuracy of these LFAs in all populations of interest. Further, local regulatory approvals, reasonable costs, and wide distribution of the tests will be needed to maximize their impact.

While molecular testing has become more widely used, there currently is no standardization and no current U.S. FDA-approved tests. Further research and standardization of PCR could lead to commercialization and/or broader use.

This diagnosis of histoplasmosis continues to require clinical suspicion, understanding of the clinical syndrome, underlying immune suppression, and overall risk to best approach the appropriate diagnostics to obtain a diagnosis. While the future of lateral flow assays and PCR testing is exciting and may improve care rapidly, a diagnosis will only be made if the clinician has a high clinical suspicion.

## 5. Conclusions

Histoplasmosis continues to cause significant morbidity and mortality globally, especially in those with underlying immunosuppression both in persons with and without HIV. While there is increased immunosuppression, there is increased testing worldwide contributing to better epidemiological data on histoplasmosis globally. Yet, while there has been expansion of the traditional areas of endemicity to more up to date global maps of endemicity, our understanding of the geographic distribution of histoplasmosis is undoubtedly incomplete and diagnostic test penetration needs to improve significantly concurrent with diagnostic test development to maximize advances in diagnostic testing.

## Figures and Tables

**Table 1:** Diagnostic tests performance and uses in histoplasmosis.

Test	Sensitivity *	Specificity *	Strengths	Weaknesses
Culture [96]	40–74%	Inadequate data	More useful in SPH and CPH	Slow growth, up to 5 weeks
Histopathology [96,97]	9–50%	Inadequate data available	Rapid results (hours)	Required highly skilled personnel, invasive tissue samples
Antigen tests [26,98,99,100]	82–95%	97–99%	Accessible, high sensitivity. Serum or urine options.	Cross-reactive with other fungi Low accuracy for subacute pulmonary form
Antibody tests [94,95,101,102,103]	54–95%	58–100%	Fast results, within days	Decrease utility in HIV, IS. Demonstrates exposure, not necessarily active infection. Cross-reactive with other fungi
PCR [104,105]	86–98%	99–100%	Fast results, within days	No standardization or commercially available assays
Beta D glucan [92,93,106]	53–92%	68–97%	Accessible, high sensitivity (except CSF)	Non-specific

* Test performance varies by patient immune function and site of infection. In general, microbe-focused tests perform best in persons with HIV and in those with disseminated disease due to higher fungal burden. Antibody testing generally performs better in sub-acute chronic disease compared to acute illness. Subacute pulmonary histoplasmosis (SPH), chronic pulmonary histoplasmosis (CHP), cerebrospinal fluid (CSF), Human Immunodeficiency Virus (HIV), immunosuppression (IS).
